# Authors’ Response to Peer Reviews of “Representing Physician Suicide Claims as Nanopublications: Proof-of-Concept Study Creating Claim Networks”

**DOI:** 10.2196/40158

**Published:** 2022-07-01

**Authors:** Tiffany I Leung, Tobias Kuhn, Michel Dumontier

**Affiliations:** 1 Care and Public Health Research Institute Maastricht University Maastricht Netherlands; 2 Department of Internal Medicine (adjunct) Southern Illinois University School of Medicine Springfield, IL United States; 3 Department of Computer Science Vrije Universiteit Amsterdam Amsterdam Netherlands; 4 Institute of Data Science Maastricht Netherlands

**Keywords:** physician suicide, suicide, suicide prevention, physician well-being, physician mental health, nanopublication, physician, doctor, mental health, semantic publishing, bibliometrics, claim network, information distortion, misinformation

*This is the authors’ response to peer-review reports for “Representing Physician Suicide Claims as Nanopublications: Proof-of-Concept Study Creating Claim Networks.”*

## Review Round 1

### Anonymous [1]

Comment: This paper [2] proposes a citation network of scientific publications about physician suicide. Such a citation network is a pioneering work for examining accurate claims of physician suicide. The network idea and entity schema design present unique values toward understanding the challenge.

1. Information completeness concerns: the authors claim that “A subset of articles from the literature search were identified that made an assertion (claim) about the annual rate of US physicians who die of suicide. Additional articles published between August 2019 and March 2020 have been identified and manually added to the article set used for this study.” However, such a data-searching procedure is not comprehensive and may lead to biased research. For example, the same source [3] of the Accreditation Council for Graduate Medical Education cited a paper back in 2003 with the same number, 300. If I did a google search or a professional database, I can find many more beyond the selected time periods. I would argue such an approach has a strong time bias and source bias. Do the authors conduct the investigation on a reliable database?

Response: Thank you for this observation. As noted in the manuscript, the data source for articles that asserted the claim of interest was a previously published scoping review of the literature about physician suicide. Regarding the web search, this manuscript used only articles published in peer-reviewed literature as an original data source for a claim about the annual suicide rate. Web search was out of scope for the retrieval step of this proof-of-concept study. These points have also been clarified in the Methods, and a statement was also added to the Limitations to indicate that a web search of the claim could provide additional insights regarding misinformation propagation of the physician suicide claim on the internet.

A revision in the Methods has detailed this approach for clarity and so that the reader has greater detail of the search strategy used: “Briefly, in that literature review, a medical librarian assisted in refining the research question, developing the search strategy, and conducting a search of relevant electronic databases, including Ovid Medline, PsycINFO, and Scopus. These databases were searched from inception through March 2020 and using the predefined literature review methodology, 347 articles were identified for analysis, with the earliest dating back to 1903 [​7]. From these 347 articles, articles were further screened for this proof-of-concept study to focus on articles that made an assertion, or claim, about the annual rate of US physicians who die of suicide. Additional articles from peer-reviewed journals were published through March 2020 were identified and added to the article set used for this study. Websites, news articles, blogs, white papers, organizational or institutional reports, and other gray literature were not the focus of this study and therefore not retrieved for inclusion as original sources of the annual suicide rate claim.”

A revision in the Limitations notes is as follows: “Finally, there may be a limitation based upon the search strategy that contributed to the data source used for this study. As web search may also offer a valuable source of nonpeer-reviewed literature and gray literature that also make a claim similar to ‘300 to 400 US physicians die by suicide annually’; these may offer an unstudied area of misinformation in public-facing publications about physician suicide. As this study was not designed as an infodemiology study, however, incorporating such a search to enrich the data source and further analysis could add to the current literature about physician suicide.”

Comment: 2. Nanopublication schema design: the schema is not well designed. For example, Figure 1 shows the number of fields is fixed and nonextensible. Therefore, that will lead each nanopublication to a limited citation size and a biased network. The authors may consider collaborations with scientists in a database or in computer science to redesign the toolkit. In addition, nanopublications can be revised or removed, and this design may lead to many false submissions. The authors may need to think about how to approach this because one contribution of this work is the toolkit.

Response: We appreciate the comment of the reviewer, although respectfully note that the template used is not a complete reflection of our abstract schema. The template, itself being represented as a nanopublication, can easily be extended and improved. It is therefore not “fixed and nonextensible.” If instead the intent of the reviewer was to note that “publishing nanopublications cannot be undone” (instead of “... can be undone”), then we note that nanopublications can be retracted, such that they are no longer shown in a regular setting but can still be accessed as an archived version.

Comment: 3. Some links are not accessible in the manuscript, such as [4].

Response: Thank you for noticing this. We apologize for this error. There was a “-“ that should not have been present in the link, which caused it to be inaccessible for the reviewer. This has been corrected.

Comment: 4. The figures (eg, Figure 1) in the documents are quite blurry. The authors should consider using pictures with high resolutions.

Response: Thank you for noting this issue from the preprint version of the manuscript. We have uploaded higher-resolution versions of all figures in accordance with JMIR Publications’ author instructions.

### Anonymous [5]

Comment: This paper tries to address a myth that has been and continues to be perpetuated around the number of US physician suicides. It tries to put forward an approach to addressing myths in a way to better inform specific populations (ie, in this case, physicians) of the reality of suicide in the profession.

1. This is an important effort and should be applauded.

2. This confirms that the myth remains in existence, despite minor changes to it, and should be either verified or dispelled.

3. The approach presented to address situations as described is one that has merit and can be useful to many where there is interest and desire. The paper was well written, clear, data driven, and of a good length. I would have liked a bit more in the discussion section (eg, implications could be stronger) and a simple conclusion statement at the end.

Response: Thank you to Anonymous [5] for the positive and supportive comments. We also agree that it is important to be able to readily identify misinformation, especially regarding a topic such as physician suicide. We believe that this study offers a new perspective on an important topic and an opportunity to potentially apply a similar schema to understanding misinformation propagation in claim networks about other topics. We have revised and reorganized the Discussion section for content and to match JMIR Publications style.

### Anonymous [6]

Comment: This paper integrates these various claims, enables the verification of nonauthoritative assertions, and makes informed statements in the advocacy of physician suicide prevention, thereby better equipping researchers and advancing evidence-based knowledge.

Response: Thank you to Anonymous [6] for the kind and encouraging comments. We agree this is an important area of study and an opportunity to apply new technologies toward understanding the field of physician suicide.

### Reviewer AF [7]

Comment: This study describes the use of nanopublications as a means to create a citation network of claims. The authors suggest that this approach allows for verification of claims in scientific literature. In the case of this particular article, the authors describe a process in which nanopublications are created from assertions of physician suicide incidence and describe their findings. Notably, the authors report that “the network is not fully connected,” “no single primary source of the claim could be identified,” and “all end-point citations either had a claim with no further citation, had no apparent claim, or could not be accessed to verify the claim.”

I believe this work is important for the methods used and the purpose of the study more than it is for the actual finding itself (which is also important). Properly implemented, the approach used could be very important in improving the validity of claims cited in scientific literature. As demonstrated in this study, it is important that assertions be verifiable in order to prevent the propagation of misinformation or distorted information. The propagation of misinformation can impact future work, as the assertions may influence the way future researchers pursue investigation. Furthermore, misinformation or inaccurate information in peer-reviewed literature can negatively impact the perceived integrity of the scientific process. As such, I believe the methods used by the authors deserve attention but should also be examined carefully to ensure the way in which this approach is implemented is thorough and can accurately identify the primary source of claims if possible.

The authors do an excellent job of describing the purpose of their work and provide the spreadsheet used to create the nanopublication index. This is helpful in evaluating the work and ensuring accuracy. Given the importance of this work, one aspect of the methodology is unclear and, in my opinion, should be made clear before the article could be considered suitable for publication.

1. The process to determine how an assertion was cited (if at all) is unclear. Optimally, any statement providing quantitative information, such as the one investigated in this study, should be directly followed by the relevant citation. This is not always the case, however, especially when multiple statements are made based on the same source and especially if they build on each other. If the authors only consider citations immediately following the assertion, they may have missed the reference provided shortly prior, or at the end of the paragraph. It would be helpful if the authors provided additional detail on this process, so that this process can be applied more consistently by other research teams using this approach.

Response: We appreciate the positive comments of Reviewer AF [7], as well as the request for clarification regarding the procedures used for collecting each of the claims from the manuscripts. These clarifications have been added to the Methods. All sources of the claim were read in full by the first author to ensure that no additional related information was missed from each of the sources of the claim. Additionally, another reviewer inquired about whether snowballing reference lists was used; this was addressed in the revision of the Methods section and is acknowledged as a potential limitation of the current proof-of-concept study in the Discussion section.

Comment: While I commented on the process in which they determine which citations to evaluate, I have also examined the articles in Figure 2 for which no reference was provided. The only potentially missed reference was a 1977 JAMA article by Sargent et al [8]. Regarding that paper, the relevant statistic (300-400 physicians per year) is not mentioned anywhere. Overall, I am not concerned about the thoroughness of the process used but advise that the exact details be included in the methods.

Response: Thank you for this comment. The Methods have been significantly revised and reorganized both to address the reviewer’s request for additional details as well as to match JMIR Publications style. The 1977 article by Sargent et al [8] is familiar to the first author and indeed does not mention the specific claim of interest. This points precisely to the original research question examining where this claim may have originated from. The Discussion, particularly the Limitations, has been elaborated to note areas for further work regarding different ways to represent data about the physician suicide incidence, which could be beneficial and foundational for further suicide research network and platform development.

Comment: Figure 2 appears to have some errors (there may be others I have not noticed; I suggest the authors review the entire figure for accuracy):

- The nanopublication links for Withy et al [9] and Anzia et al [10] are identical; it appears the link for Anzia (2016) in the figure is incorrect when compared to the excel file.
- The year of publication for what appears to be Andrew and Brenner (2018) in the excel file is listed as 2015.

Response: We thank the reviewer for their detailed review and corrections for the figure. Figure 2 is now Figure 3 in the resubmission. The nanopublication for Anzia has been corrected in the figure. Additionally, the nanopublication link for the Andrew and Brenner 2015 version of the website has been corrected in the figure.

### Reviewer AL [11]

Comment: This paper presents a study focused on “the use of nanopublications as a scientific publishing approach to establish a citation network of claims drawn from a variety of media concerning the rate of suicide of US physicians.” The study finds interesting results, and I have the following comments and concerns.

1. Consider the sentence “To our knowledge, no such application to this field has previously been done.” Authors should provide related work to argue this. Comparison with previous works is missing in the paper. Are there others related to nanopublications?

Response: Thank you for this request for clarification. There is no prior work applying nanopublications in this way to asserted claims in published peer-reviewed literature. The work by Clark [12,13] has been added and cited with regards to work on micropublications, which is not the same as the present schema applied in this study. A sentence has been added to the Introduction to acknowledge Clark’s work on micropublications: “…previous work on micropublications, which are a semantic model for scientific claims and evidence that enables knowledge discovery and inference across networks of information. A similar approach to identify and trace citation distortion had previously been done regarding a specific scientific claim about Alzheimer’s disease. The current study extends this work by applying the nanopublication schema to the same physician suicide claim.”

Comment: 2. The paper would improve if examples (at least one) of nanopublications used in the data source were added. This would be illustrative.

Response: We appreciate this comment and feedback from the reviewer. The entire first paragraph of the Results section has been added to the revised manuscript, and it now details one set of nanopublications. They are illustrated in Figure 2.

Comment: 3. Reference Leung et al [14] (2019) has been published and is apparently peer-reviewed. Check if there are other references to be added in the data source.

Response: We greatly appreciate the comment from the reviewer and note that we have clarified the data source used in this proof-of-concept study in a Data Source subsection of the Methods. The authors had already used the final reference list from the published article, not the MedRxiv preprint [15], for this study. The reference has been updated in the reference list of this manuscript.

The Data Source subsection of the Methods section is more detailed, briefly summarizing the search strategy: “Briefly, in that literature review, a medical librarian assisted in refining the research question, developing the search strategy, and conducting a search of relevant electronic databases, including Ovid Medline, PsycINFO, and Scopus. These databases were searched from inception through March 2020 and using the predefined literature review methodology, 347 articles were identified for analysis, with the earliest dating back to 1903. From these 347 articles, articles were further screened for this proof-of-concept study to focus on articles that made an assertion, or claim, about the annual rate of US physicians who die of suicide. Additional articles from peer-reviewed journals were published through March 2020 were identified and added to the article set used for this study. Websites, news articles, blogs, white papers, organizational or institutional reports, and other grey literature were not the focus of this study and therefore not retrieved for inclusion as original sources of the annual suicide rate claim.”

Comment: 4. Consider these two sentences: “A subset of articles from the literature search were identified that made an assertion (claim) about the annual rate of US physicians who die of suicide. Additional articles published between August 2019 and March 2020 have been identified and manually added to the article set used for this study.” I think these sentences should be unpacked. How were these two steps performed?

Response: Thank you for this question, which we have addressed, based on your previous comment, as well as that of another peer reviewer requesting this clarification.

Comment: 5. The main results of this paper are in Table 1, which “revealed that (1) the network is not fully connected, (2) no single primary source of the claim could be identified, and (3) all end-point citations either had a claim with no further citation, had no apparent claim, or could not be accessed to verify the claim.” This is interesting, but what was the rationale for using nanopublications as a tool in the methodology? Could these results be found using snowballing as a review method?

Response: As a proof-of-concept study, this study sought to explore the claim network for a single claim about physician suicide rates. This was the rationale and aim for applying nanopublications as the tool, and the focus was to retrieve specifically the citation noted in a published article in relation to the claim made. As this approach did not involve snowballing to manually search the full reference list of each of the included articles, we have added this to the limitations. The limitation is as follows: “Second, regarding the data source approach, snowballing to examine full reference lists of the included articles was not performed. The focus for this proof-of-concept study was to specifically focus on the citation that the author of an article provides at the end of the sentence that makes the annual suicide rate claim. Snowballing may reveal additional publications that make the same claim, but we also anticipate that this approach would add further evidence that the claim network about annual suicide rate would reveal additional fragmented and disconnected parts of the network. Additional investigation would be needed to explore this hypothesis.”

Comment: 6. What are the contributions of the paper? They could be explicitly declared. Moreover, the objective of the paper should be better declared—“In this paper, we aim to create nanopublications from assertions relating to physician suicide incidence.” I think this is not the same from the abstract, which is much better.

Response: Thank you for this excellent suggestion. The aim statement in the abstract (Objectives) and in the manuscript (at the end of the Introduction), now consistently states, “The aim of this study is to use nanopublications as a scientific publishing approach to create a citation network of claims in peer-reviewed publications about the rate of suicide of US physicians.” A similar comment made by another reviewer regarding clear statements about the paper’s contributions has led to revisions of the Discussion and Conclusion. We have revised and reorganized the Discussion section for content and to match JMIR Publications style.

Comment: 7. and 8. [a] eg to eg, (add comma); [b] et al to et al. (add dot)

Response: These have been corrected. We look forward also to JMIR Publications’ copyeditors assisting further with any additional stylistic changes needed to conform to the journal’s publication standards.

Comment: 9. Figure 1 is in low quality.

Response: Thank you for noting this issue from the preprint version of the manuscript. We have uploaded higher-resolution versions of these figures in accordance with JMIR Publications’ author instructions.

Comment: Remove “-” from URLs:

http://purl.org/np/RAqWlNPJt3Eb4HkmPCpjaiR“-” HGCzKIZag6cBNMkG8nxu6I

Response: Thank you for noting this issue, as did another reviewer. This was unfortunately an artifact that arose from the different formatting used in the preprint version of this manuscript. This has been corrected in the revised submission.

## Review Round 2

### Reviewer AF [7]

#### General Comments

The authors appear to have addressed many of the concerns raised by me and by other reviewers, but some comments still have not been addressed satisfactorily. I do feel this work is important, but the below comments should be addressed before publication.

#### Specific Comments

##### Major Comments

Comment: Other reviewers brought up the statement “Additional articles published between August 2019 and March 2020 have been identified and manually added to the article set used for this study.” While I believe the authors clarified a separate concern raised by one of the authors, this statement requires additional clarification. It is unclear how those articles were identified, and this should be explained.

Response: Thank you for repeating this comment to ensure we have provided sufficient methodologic detail to address the previous comment more thoroughly. This has been addressed in two ways in the revised manuscript. First, the Methods section has been revised with the requested detail during revision (the revised text is also included below). Second, the spreadsheet containing the data set that is openly accessible on FigShare [16] has been updated with an additional column to note where each reference originated from.

“One author (TIL) established a Google Scholar alert using the keyword, ‘physician suicide,’ and screened additional articles from peer-reviewed journals to include based on earlier established inclusion criteria from the published scoping literature review [​7]. …The spreadsheet also notes the original source of the reference for the set: scoping literature review, Google Scholar alert, or not applicable as the citation is included because it is referenced by another claim.”

Comment: The authors appear to have misunderstood my following comment: “The process to determine how an assertion was cited (if at all) is unclear. Optimally, any statement providing quantitative information, such as the one investigated in this study, should be directly followed by the relevant citation. This is not always the case, however, especially when multiple statements are made based on the same source and especially if they build on each other. If the authors only consider citations immediately following the assertion, they may have missed the reference provided shortly prior, or at the end of the paragraph. It would be helpful if the authors provided additional detail on this process, so that this process can be applied more consistently by other research teams using this approach”.

To clarify, I would like more details on how it was determined which sources were cited to support a claim. For example, if a paper contained a paragraph with the assertion in question, it may not always have the relevant citation at the end of the statement. Take the following hypothetical statement (not from any actual paper, but for illustrative purposes):

“Physician suicide remains an important topic related to the health status of the workforce, but previous studies indicate that there are little data on the subject in the scientific literature (references 1 to 4). 300 to 400 US physicians die by suicide annually, and a recent economic analysis estimates that physician suicide results in the loss of US $XXXXX per year from the American health care system (reference 5). Consequently, physician suicide is the *Y*th cause of death among physicians (reference 6).”

The “300 to 400” physician number should be found in reference 5, but that is not always the case, especially if later edits were made and the change was not noticed. Sometimes, there is no citation “5,” and the statistic is derived from references 1-4 or 6. While this is obviously not good practice, it does occur in scientific papers occasionally and not infrequently in other types of publications (while I cannot remember for sure, I believe one of the studies cited is missing a citation similar to 5). I would like to clarify if the authors attempted to account for other proximal references, which is different from snowballing, but arguably may catch sources that otherwise could be missed.

Response: Thank you to the peer reviewer for the specificity in clarifying this particular comment. We have revised the Methods section to include a Data Extraction subsection to address the reviewer’s comment more completely. The revised subsection includes the following text in the revised manuscript:

“To ensure that citations provided to support a claim were sufficiently identified, the sentence preceding and following the claim of interest were checked for a citation. Textbox 1 illustrates an example of the extraction procedure on the level of the manuscript and claim.

Textbox 1. Claim identification and attribution during data extraction.” Please see the revised manuscript for the full textbox.

With regards to the peer reviewer’s comment that “Sometimes, there is no citation 5, and the statistic is derived from references 1-4 or 6. While this is obviously not good practice, it does occur in scientific papers occasionally and not infrequently in other types of publications…” we also hypothesize that this may be a commonly occurring issue in the area of physician suicide; however, this is beyond the scope of this paper. We acknowledged this already in the Limitations section of the manuscript, noting that this proof-of-concept study focuses only on a verbatim claim, and that “Further work is needed to represent all available data on physician suicide, beyond focusing on the single claim studied here. Representing additional data as nanopublications, including incidence data, risk factors, demographics, and other contextual information, may offer an even richer graph of existing knowledge about physician suicide to enable more rapid learning about the field.”

### Reviewer AL [11]

Comment: I congratulate the authors for their work. All my questions were answered, and concerns addressed. Thank you!

Response: Thank you to the peer reviewer kindly for the thoughtful and detailed peer review.
